# Design of a Website on Nutrition and Physical Activity for Adolescents: Results From Formative Research

**DOI:** 10.2196/jmir.1889

**Published:** 2012-04-26

**Authors:** Debbe Thompson, Karen Weber Cullen, Carol Boushey, Karen Konzelmann

**Affiliations:** ^1^USDA/ARS Children's Nutrition Research CenterDepartment of PediatricsBaylor College of MedicineHouston, TXUnited States; ^2^University of Hawaii Cancer CenterEpidemiology ProgramUniversity of HawaiiHonolulu, HIUnited States; ^3^Organizational and Educational ConsultantPearland, TXUnited States

**Keywords:** Internet, intervention, obesity prevention, food, physical activity

## Abstract

**Background:**

Teens do not meet guidelines for healthy eating and physical activity. The Internet may be an effective method for delivering programs that help them adopt healthy behaviors.

**Objective:**

To collect information to design content and structure for a teen-friendly website promoting healthy eating and physical activity behaviors.

**Methods:**

Qualitative research, encompassing both focus group and interview techniques, were used to design the website. Participants were 12-17 year olds in Houston, Texas, and West Lafayette, Indiana.

**Results:**

A total of 133 participants took part in 26 focus groups while 15 participated in one-on-one interviews to provide guidance for the development of teen-friendly content and structure for an online behavior change program promoting healthy eating and physical activity to 12-17 year olds. The youth made suggestions to overcome common barriers to healthy eating and physical activity. Their feedback was used to develop “Teen Choice: Food & Fitness,” a 12-week online behavior change program, populated by 4 cartoon character role models.

**Conclusions:**

It is critical that members of the target audience be included in formative research to develop behavior change programs that are relevant, appealing, and address their needs and interests.

## Introduction

Youth obesity has reached epidemic proportions [1] and adolescence appears to be a particularly critical intervention period [2,3]. Current obesity prevention interventions, such as those that are school-based, have generally not improved body composition outcomes [4] suggesting different approaches are needed to impact obesity risk.

To be effective, behavior change interventions must meet the expectations of today’s technology-savvy users [5]. Online programs allow wide access to interventions, and the use of graphics and Web applications with interactivity allow the user to actively participate in programs that can promote healthy choices [6]. Reviews of the literature have identified that online interventions for adults have shown some success [7-12]. Youth online interventions have also been somewhat successful at improving diet [13-16] and physical activity [13,16,17] behaviors, and promoting weight loss [18]. Critical components for success in these endeavors appears to be the active participation of youth in the development of such programs [6,19] and the use of theory and behavior change techniques to guide program development [20].

Although there has been concern regarding youth access to computers and the Internet among the general population, data reveal a somewhat different picture. Nationally representative surveys of media use reveal that home computer access among 8-18 year olds ranges from 89% to 94%, while 74% to 88% have home Internet access [21]. Approximately 55% of the adolescents surveyed reported searching for health information on the Internet [21], while approximately 63% of youth aged 12-17 years reported daily Internet use [22]. Further, the number of youth with high-speed Internet access at home has increased [21,23]. Therefore, online behavior change programs promoting healthy eating and physical activity behaviors have the potential to reach large numbers of youth in a familiar, convenient, and readily available manner. This paper presents the results of formative research that was conducted to inform the design of such a program for youth between ages 12 to 17 years.

## Methods

Focus groups and one-on-one interviews were conducted to inform the content and structure of the online program, as well as to evaluate its components during development. The study was approved by the institutional review boards of Baylor College of Medicine in Houston, Texas, and Purdue University in West Lafayette, Indiana. Adolescents aged 12-17 years were recruited from youth centers and schools using standard recruitment techniques. Participants provided written parental consent and their own verbal assent prior to participation. Teens could participate in only one activity (ie, one focus group or one interview).

### Focus Groups

Trained moderators and assistant moderators [24] conducted focus groups in community settings in Houston, Texas, and West Lafayette, Indiana. Focus groups were conducted in two different states to ensure the information obtained was not just relevant to teens in one area of the country. Focus group moderators followed a semi-structured script, and probes and prompts were used to expand and clarify responses. The moderator led the discussion and an assistant moderator recorded responses. Separate focus groups were held for nutrition and physical activity in order to develop content. Additional focus groups were held to adapt an existing healthy eating calculator to make it teen-friendly.

#### Healthy Eating and Physical Activity

The focus groups covered two general areas: (1) what youth thought should be included in an online program about healthy eating or physical activity and (2) suggestions for overcoming common barriers to healthy eating or physical activity. The information obtained on barriers and suggested solutions was used to create short role model video clips to be included in the online behavior change program under development.

To spark the discussion, separate lists of commonly reported barriers were created for healthy eating [25-41] and physical activity [42-58] (Table 1). As participants arrived for the focus group, they were given the list of diet or physical activity barriers (depending on the focus of the particular group) and were asked to check off those items on the list that were problems for them. The sheets were collected at the beginning of each focus group and the results were summarized on a large poster board. The 5-7 barriers that received the most votes were used to guide the discussion on ways to overcome each barrier. In addition, teen cartoon characters (ie, the role models who would appear in the program) and potential names for the online behavior change program were vetted by the focus group participants. After each focus group, the moderator and assistant moderator discussed the results (ie, debriefed) and generated a report that summarized the important findings from that group.

**Table 1:** Percentage of responses to commonly reported barriers to healthy eating [25-41] and obtaining physical activity [42-58] from adolescents attending initial focus groups.

Barriers		%
**Barriers to healthy eating**		
	They eat too many snacks.	69
	They like to eat foods from school vending machines or snack bar.	67
	They drink a lot of sodas or sweetened beverages every day.	61
	They skip breakfast.	54
	They don’t like most healthy foods, such as vegetables.	35
	Their friends tease or make fun of them if they eat healthy foods.	35
	Few or none of their friends eat healthy foods.	34
	They eat at restaurants a lot (eg, several times a week).	31
	Their friends do not eat healthy foods at restaurants.	29
	They don’t know how to prepare foods.	27
	They think healthy foods cost too much.	23
	Unhealthy foods taste better.	22
	They don’t like healthy foods in general.	11
	It takes too much time to make healthy foods.	9
	They do not have healthy foods at home.	5
**Barriers to physical activity**		
	They would rather do other activities (eg, play video games, watch TV, or talk on the phone).	65
	They have too much homework.	59
	Their friends do not like or do physical activity with them.	59
	They are too busy with afterschool activities or chores.	41
	Bad weather (eg, rain, snow, or heat) is a problem.	39
	They do not have enough time to be physically active.	31
	They are not good at most physical activities.	27
	They think physical activity is too hard.	21
	They do not have physical activity equipment at home.	21
	They don’t have the money to pay for sports or physical activity costs (eg, clothing, equipment, or fees).	19
	They do not have a safe place to be physically active.	14
	They can’t get to practices or places to be physically active.	14
	No one reminds them to be physically active.	13
	They think physical activity makes their body hurt.	12
	They don’t like to sweat.	11
	They worry that other people will laugh or tease them when they do physical activity.	9
	They think physical activity messes up hair and/or makeup.	4

#### Healthy Eating and Physical Activity Calculator

Additional focus groups were conducted to develop an online “calculator” that would be easy to use, in a format acceptable to this age group, and that provided information requested by the adolescents on what and how much to eat and how much physical activity they needed to do. An existing healthy eating calculator developed for adults was used to prompt discussion.

#### Program Development

Using information obtained from the focus groups, investigators created the online program components. Interviews were then conducted to review the materials prior to finalizing the program.

### Interviews

Interviews (conducted in Houston only) followed a semi-structured script. Probes and prompts were used to expand and clarify responses. Print versions of the teen cartoon characters (ie, online role models) and components of the behavior change program were used to guide the discussion. Two types of interviews were conducted: one set was to review role model scripts and teen characters; the second set of interviews was to review online behavior change program content and structure prior to finalizing the program. Participants for the first set of interviews were recruited from the same locations as the focus group participants using procedures described above. The second set of interviews was promoted only at the Children’s Nutrition Research Center in Texas and interviews were conducted with children, relatives, and/or neighbors of faculty and staff.

## Results

### Focus Groups

In total, 18 initial focus groups—10 on healthy eating (n = 50 participants) and 8 on physical activity (n = 45 participants)—were conducted (Table 2). Participants stated that there should be an initial log-on page where each person could pick an avatar (ie, an online teen cartoon character to serve as a digital guide in the program) [59] and the log-on page should also provide ready access to all the different Web pages available in the behavior change program. Information on basic nutrition and physical activity concepts was also requested, as was information on what they should eat (ie, what foods were healthy) and how much physical activity they should get each day. They also liked the idea of goal setting and problem solving, as well as having a goal sheet that included a list of goals from which they could choose, a plan of action, and a self-monitoring form. Goal review should appear at subsequent log-ins. A blog was thought to be an important component, but only if entries were prescreened before posting. Teens who participated in the healthy eating focus groups also recommended including healthy recipes for themselves and their parents.

The top barriers to healthy eating and physical activity suggested by the groups are listed in Table 3. These barriers were used to create the role model stories. Online behavior change program components suggested by participants were consistent across focus group type (healthy eating or physical activity) and location (Texas or Indiana).

An additional 8 focus groups (n = 38) were conducted to develop a healthy eating and physical activity “calculator” to provide tailored information on nutritional needs and physical activity. Participants suggested a graphic “pie chart” figure that would provide both textual and visual information. They also suggested that users should be able to click on the various “wedges” of the pie to access additional information about that component. This design would give participants control over how much and what information they accessed.

**Table 2:** Characteristics of the content development focus group participants (N = 95 participants in 18 focus groups)

Characteristic		n (%)
**Gender^a^**		
	Male	47 (50)
	Female	47 (50)
**Race/Ethnicity^b^**		
	African-American	10 (11)
	Hispanic	36 (38)
	Other	3 (3)
	White	46 (48)

^a ^Information about gender was unavailable for 1 participant.

^b ^Participants were asked to respond to these racial/ethnic categories only.

**Table 3:** Top barriers reported in focus groups and the videos created to address each barrier.

Barriers			Video title
**Healthy eating barriers**			
	1	Snacks	*Handle your Snack Attack*
			*Nothing to Eat*
	2	School foods	*See What’s Cookin’ at School*
	3	Soda/sweetened beverages	*Drop the Pop*
	4	Breakfast skipping	*Energize your AM*
	5	Friend influences	*Eat Healthy on the Go*
	6	Restaurant eating	*Eat Healthy on the Go*
			
**Physical activity barriers**			
	1	Like other activities better	*Choose to Move*
			*Get Moving!*
	2	No time	*Juggle Time: Fit it all in*
			*Fit Fitness in your Day*
	3	Friend influences	*Friends Make Fitness Fun!*
	4	Weather	*No Excuses: Movin’ at Home*

### Interviews

#### Role Model Stories and Teen Characters

Using information from the focus groups that addressed common barriers to healthy eating and physical activity and how to overcome them (Table 3), 12 role model stories with 4 teen cartoon characters (ie, online role models) were identified. The scripts of the role model stories were written by a professional writer and reviewed by adolescents (n = 10) in individual interviews prior to finalizing them to ensure youth appeal. Interviews indicated that the youth liked the stories and the teen cartoon characters. An important change suggested during the interviews was that the teen cartoon characters store photos on their cell phones, laptops, or on social networking sites rather than in a photo album as portrayed in one of the role model stories.

#### Online Behavior Change Program Content

Using information from the focus groups and interviews, program components for the online program were developed. A final set of interviews (n = 5) was conducted to review program components prior to completion. During these one-on-one interviews, adolescents were shown the components and queried about their thoughts regarding relevance, appropriateness, and appeal. The interviews indicated no changes were needed to program content.

### Final Online Behavior Change Program Structure and Content

**Figure 1:**
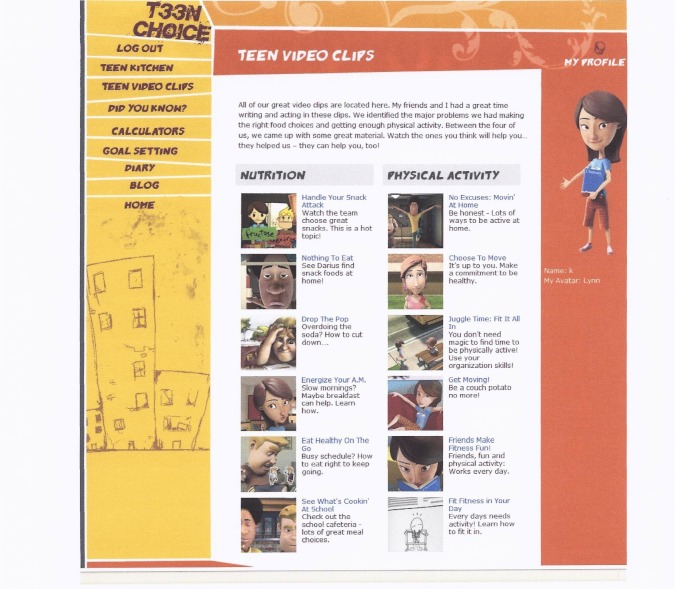
Screenshot of "Teen Video Clips" (short, animated role model stories) showing titles of topics addressed in the online behavior change program).

**Figure 2:**
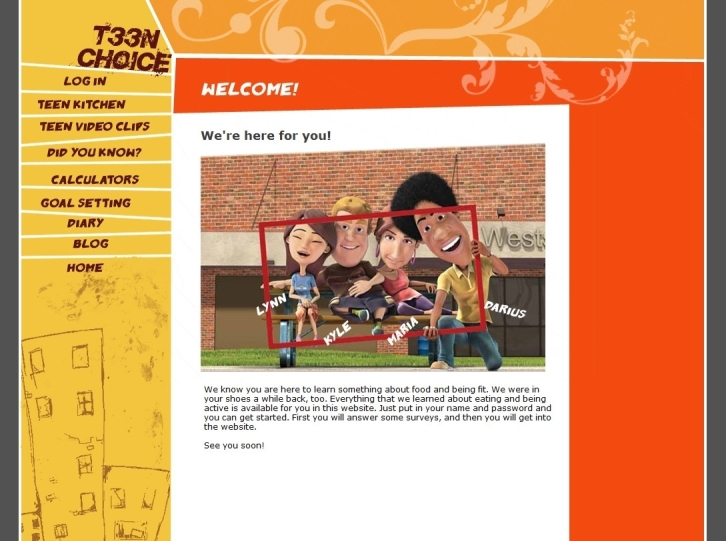
“Welcome” screenshot showing the online teen characters (ie, role models).

**Figure 3:**
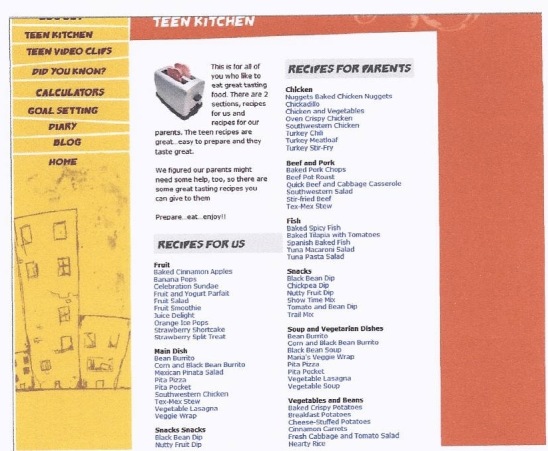
“Teen Kitchen” screenshot showing teen and parent recipes.

**Figure 4:**
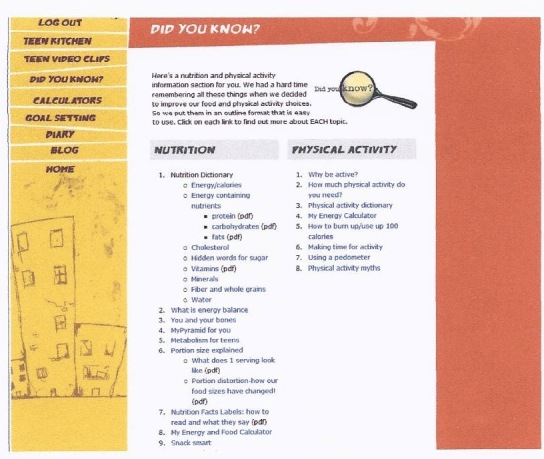
“Did You Know” screenshot showing topics.

**Figure 5:**
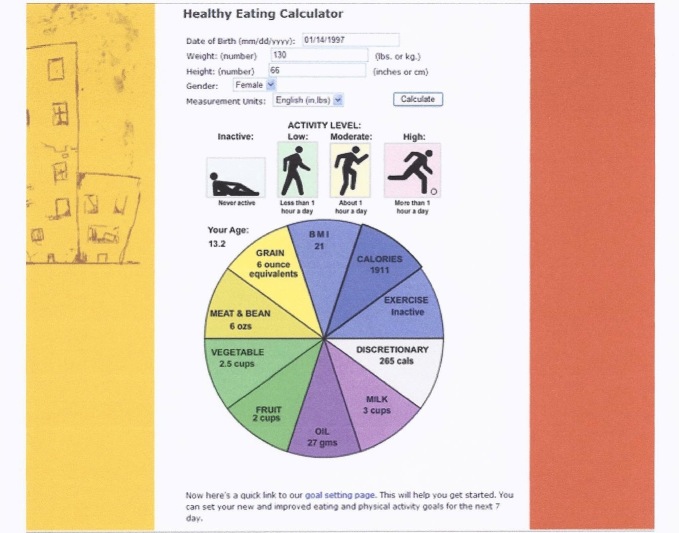
“Healthy Eating Calculator” screenshot.

The name selected by participants for the 12-week online behavior change program was “Teen Choice: Food & Fitness.” The online program contains an initial log-on page where teens enter their unique username and password to log on to the program website that is hosted on a secure server. Once on the website, they can view the 12 role model stories addressing barriers to healthy eating (n = 6) and physical activity (n = 6) (Figure 1) led by 4 teenage cartoon characters (ie, role models) (Figure 2). Figures 3 and 4 show screenshots of the recipes for teens and parents (“Teen Kitchen”) and the nutrition and physical activity information sections (“Did You Know?”) created in response to focus group discussions. Figure 5 shows the healthy eating calculator. In addition, teens can access a refereed blog, set goals, make plans to help them attain their goal, track their progress online, report goal attainment, and participate in problem-solving activities.

**Control Program Content and Structure**

For the randomized controlled trial to test the effectiveness of the online program at improving eating and physical activity behaviors, a control condition was needed. It was constructed by removing the role model stories and the goal setting, planning, self-monitoring, goal review, and problem-solving components from the treatment intervention (ie, the components that promote personal mastery and observational learning, two key components of Social Cognitive Theory [60]).

## Discussion

### Principal Results

This paper reports the results of formative research with adolescents to create an online program promoting healthy eating and physical activity that would appeal to this age group. Their recommendations were used to guide decisions about the online program content and structure. As such, it provides guidance on how to involve the target audience in the development of an online behavior change program.

### Comparison With Prior Work

Internet and computer use are high among youth [21], thus offering a potential method for reaching them in a manner that is familiar, appealing, and readily available. The involvement of youth in the creation of online programs aimed at helping them reduce obesity risk, such as those that promote healthy diet and physical activity behaviors, is critical [61,62]. Although emerging evidence suggests online programs may be an effective method for modifying youth health behavior [13,14,16,17,63-66], few programs have been developed specifically for adolescents [14,16,17,65,66]. Thus, this paper offers a model for development of online programs for adolescents that demonstrates how to involve them in the design process.

Online programs provide an engaging venue for achieving behavior change through both personal mastery and observational learning. Personal mastery can be promoted in an online program through inclusion of self-regulatory activities, such as goal setting, planning, self-monitoring, and problem-solving activities; thus, it is important for these activities to be developmentally appropriate and utilize a format that appeals to the target audience [67]. Additionally, it is imperative that the content appeal to youth and reflect their reality. For example, the barriers to healthy eating and physical activity identified by youth reflect those specified in the literature [25-58]; however, it was critical to identify their top barriers and to create role model stories that reflected solutions perceived as realistic by teens. Formative research, such as that reported here, can provide important insights that are critical to achieving this goal.

Observational learning occurs by watching others (ie, role models) perform a particular behavior and receive rewards [60]. This process is facilitated when the role model is perceived to be both competent and similar to the observer [68,69]. In online behavior change programs, teen cartoon characters can function as role models [70]. Therefore, in programs attempting to capitalize on observational learning, engaging adolescents in the design of appealing and believable teen characters is essential to creating an effective program.

Attracting and maintaining attention is an important first step in observational learning because it initiates learning and behavior change processes [60]. Enhancing personal relevance [8,71] of the program components achieves this goal by alerting the participant that “this is for me.” In the current program, formative research provided an opportunity to identify and understand real and perceived barriers adolescents encounter when attempting to make healthy diet and physical activity choices, as well as solutions that made sense to them. Incorporating these barriers and solutions in role model stories provided a venue for conveying this information to teens in an entertaining, personally relevant manner.

### Next Steps

Recruitment for the randomized controlled trial to test the online program began in late 2009 and concluded in October, 2011. Data collection is currently underway.

### Recommendations/Suggestions

Design of online programs promoting behavior change should be a collaborative effort between researchers and members of the target audience, such as teens. Suggestions for achieving this are:

1. A realistic timeline is essential. Allow ample time for recruitment, data collection, analysis, interpretation, and application.

2. Participants should represent the target population. This includes gender, socio-economic status, age, and other salient characteristics. If not, it is possible the data may be skewed and may not adequately represent the target group, thus reducing the potential effectiveness of the program. In this program, formative research was conducted in two states in order to ensure the results were not relevant to teens in only one part of the country.

3. Conduct enough focus groups or interviews to achieve theoretical saturation, or the point at which no new information emerges [24]. In the program described in this paper, both focus groups and interviews were conducted to ensure the topics were adequately covered.

4. Although scripts are important for consistency in data collection, they should be semi-structured, allowing ample room for participants to share thoughts and opinions. Discrepant information (ie, data different from those heard from others) may be especially useful. Scripts should contain open-ended, neutral, and non-leading questions. Probes, prompts, and follow-up questions should be generously used to expand and more fully understand responses. The scripts used to guide discussions in the current project were semi-structured, and probes and prompts were used to explore responses and elicit additional information as needed.

5. Member checks are important [72,73]. Member checks help ensure the data are being interpreted correctly. Although there are several ways to conduct member checks, one way that is particularly useful in the design of behavior change programs is to take the results of the analyses back to members of the target audience and ask if the data were correctly interpreted and/or applied. For example, in the current study we asked youth to review the role model stories.

### Limitations

Limitations of this research include the use of qualitative research in only two locations, which limits generalizability of the findings. This research does not address desired frequency of user access, program components accessed, or time spent viewing or completing the various components. It also does not address the impact of the program on diet or physical activity behaviors. However, the outcome evaluation, which is currently underway, is collecting information to address these issues. Additional research is needed to understand triggers for participation (ie, why teens enroll in online behavior change programs), whether programs of this type meet their expectations, and long-term health effects in order to develop robust and effective online behavior change programs.

### Conclusion

Successful programs that encourage adolescents to adopt healthy diet and physical activity behaviors are needed to reduce obesity risk. Online behavior change programs designed in conjunction with youth may provide an important venue for achieving desired changes in these behaviors. The efficacy of this approach with adolescents is currently being tested and will provide valuable insights that can be used to guide future intervention research.
